# “Community strengthening through citizen monitoring of water quality: A systematic review”

**DOI:** 10.1371/journal.pone.0305723

**Published:** 2024-07-19

**Authors:** Edith Dominguez-Rendón, Mariana Villada-Canela, Dalia Marcela Muñoz-Pizza

**Affiliations:** 1 Doctorado en Medio Ambiente y Desarrollo, Instituto de Investigaciones Oceanológicas, Universidad Autónoma de Baja California, Ensenada, Baja California, México; 2 Instituto de Investigaciones Oceanológicas, Universidad Autónoma de Baja California, Ensenada, Baja California, México; 3 Profesora Titular, Facultad de Ciencias, Universidad Autónoma de Baja California, Ensenada, B.C., México; Pontifícia Universidade Católica do Paraná: Pontificia Universidade Catolica do Parana, BRAZIL

## Abstract

Citizen participation in decision-making is a fundamental democratic pillar of democracy. However, the degree of citizen involvement and recognition by governmental institutions may be conditioned by the level of competence and knowledge demonstrated. Therefore, carrying out collective projects can contribute to strengthening citizen engagement in water management issues. Nonetheless, there is limited knowledge about the various types of citizen engagement and the practices that have facilitated greater inclusion in decision-making regarding water resources. This study aims to identify and analyze practices that strengthen water community organizations through citizen monitoring-based involvement. A systematic literature review was conducted using the PRISMA method. The research was guided by the following questions: What are the differences in the level of citizen involvement and the degree of transformation facilitated by citizen monitoring of water quality (CMWQ) between the global North and South? What practices strengthen community organizations in decision-making based on CMWQ? Moreover, what challenges do community water organization structures community water organization structures face when based on CMWQ? A total of 161 publications were identified for the analysis of critical themes. After applying the eligibility criteria, 33 documents were selected for content analysis. The reviewed monitoring exercises indicate that the highest level of citizen organization achieved by participants is commonly recognized as “Water Committees”. One practice that strengthens these committees is the generation of collaboration agreements among different types of allies, based on a shared objective. However, in the Global South and at the institutional level, there is still resistance to water quality data generated by citizen monitors, especially in regions with large-scale open-pit mining projects. To sustain the efforts of community figures, monitoring programs with public funding need to be established, and public policies supporting these initiatives at the institutional level must be implemented.

## Introduction

Globally, the pressure on water resources has been increasing, resulting in diminished water availability and quality. Various strategies, such as water treatment technologies technologies focused on water treatment, conservation efforts, nature-based solutions, and water savings measures, have been implemented to mitigate the adverse impacts. However, the results remain inadequate, highlighting the urgent need to promote policy enhancements [1] to achieve sustainable water management. This advancement requires processes that integrate community participation in decision-making. Citizen science projects possess the potential to facilitate community integration and foster more adaptable and resilient governance approaches at the local level [2] grounded in data generation and collaborative endeavors [3].

When implemented effectively, participatory processes in water and sanitation management, can empower participants, cultivate greater social awareness of water related issues, fortify the legitimacy of interventions, and ultimately enhance the delivery and sustainable management of water services and their sustainability. However, such processes necessitate a contextually tailored design, underpinned by adequate financial resources and technical capabilities. Additionally, stakeholders must be well-informed about the political landscape to ensure successful implementation [4].

Citizen participation mechanisms are essential to foster public engagement in decision-making processes at all levels of public resource administration. These mechanisms promote accountability, transparency, inclusion, and responsiveness to the diverse needs and aspirations of citizens, reinforce community capacity, and safeguard human rights [5]. Consequently, global public policies aim to implement participatory mechanisms in water management, targeting local governments and empowering citizens to collect reliable and valuable data on water resources. However, there is a lack of regulations and authority-supported mechanisms that enable the inclusion of community organizations in water monitoring and supply activities [6].

The involvement of citizen scientists in projects and programs for knowledge production on water quality has created opportunities for utilizing scientific evidence in water resource management through various citizen participation methods in water quality monitoring. Citizen science, participatory monitoring, community monitoring, community-based monitoring, participatory action research, and community water monitoring indicate the varying levels of involvement citizen scientists can achieve before, during, and after a monitoring exercise. This participation not only addresses community demands but also generates data to enhance water management at the local level through informed decision-making [3, 7–10].

The transition from citizen scientists to community water organization structures (such as committees, associations, assemblies, and others) involves participants utilizing data to enhance living conditions in their surroundings community water organization structures [11–15] or to influence decision-making and advocate for public policies [16–18]. Strengthening the capacities as of these organizations can empower groups to formulate local water management policies [16], aligned with collective needs, enabling them to generate and implement solutions with the available resources at their disposal. This evolution underscores the potential for community-driven initiatives to address water-related challenges through informed decision-making and collective action

Extensive research, primarily conducted in North America and the European Union [7, 8, 10] has documented various benefits of citizen science initiatives in water quality monitoring. These include as increased public participation in local water quality data production [12, 19]; raising awareness of pollutant effects through the democratization of science [10, 20]; building mutual trust, confidence, and respect among scientists, authorities, and the public [11, 14, 21, 22]; gaining scientific knowledge and incorporating local or traditional knowledge, as well as increasing social capital and empowerment [10, 13, 23, 24]. However, such experiences in the Global South have rarely been documented in the international literature [3]. For instance, few initiatives have been documented in Latin American countries [25, 26] and other countries such as South Africa [27], India [28], and Kenya [29], highlighting a gap in the documentation and dissemination of citizen science practices in water quality monitoring from these contexts.

The distinction between the Global North and the Global South transcends beyond geography and economic development; it provides insight into the historical roles these regions have played in terms of resource supply and demand, affecting the quality of life of those inhabiting territories from which resources, such as minerals, are extracted through open-pit mining. These significant disparities impact citizen participation and the validation of shared processes. Consequently, it is essential to compare experiences and practices across regions to generate effective advocacy strategies for community-driven decision-making in water resource management. This involves organized efforts by community groups to collect data on water quality, emphasizing the need for collaborative efforts and supportive governance frameworks. Additionally, recognizing the common challenges faced by communities worldwide highlights the interconnectedness of these issues and the necessity for collaborative, global solutions. By fostering cross-regional dialogue and knowledge exchange, more inclusive and sustainable water management practices can be developed, empowering local communities and addressing shared concerns through collective action.

Developing capacities in community water organization structures require understanding the practices that need to be fostered, considering the disparities between models implemented across different contexts. In this regard, the following research questions were posed: What are the differences in the level of citizen involvement and the degree of transformation facilitated by citizen monitoring of water quality (CMWQ) between the Global North and South? What practices strengthen community organizations in decision-making based on CMWQ? Furthermore, what challenges do established community water organization structures whose face when their foundation is CMWQ? To address these questions, a systematic review was conducted using the PRISMA method, examining articles and book chapters that document citizen participation methods in water quality monitoring and their organized impact on water management.

The results of the review are presented in six sections: 1) Synthesis of selected studies, 2) Contrast of experiences between the Global North and South, 3) Community figures and strengthening practices, 4) Challenges for the consolidation of community figures, 5) Discussion, and 6) Conclusions.

## Methodology

### Protocol and record

The systematic review was conducted following the PRISMA 2020 guidelines [30]. The search results were recorded in a Microsoft Excel® matrix, version 2019, including title, author(s), country, year of publication, and keywords.

### Information sources

A comprehensive search was conducted across sixteen academic repositories in English and Spanish. The search was conducted from March 16^th^, 2023, to June 7^th^, 2023. The electronic search for English-language research was conducted in eleven academic repositories available: SCOPUS, SpringerLink, Web of Science, EBSCO, ELSEVIER, SAGE, JSTOR, Wiley Online Library, BioOne, Cambridge University Press, Conricyt, and DOAJ. Additionally, three Spanish-language repositories, Dialnet, Redalyc, and ResearchGate, were consulted to identify open access articles. Furthermore, Google Scholar was utilized to broaden the review and include relevant literature in both languages

### Search strategy

The search involved the use of key terms identified with the research questions and related concepts: methods of citizen participation (citizen science, participatory monitoring, community monitoring, community-based monitoring, participatory action research, and community water monitoring), concepts related to community water organization (volunteers, citizen scientists, water management, governance, collaborative governance), with a focus on Water Quality Monitoring. Search strings were utilized across all databases, employing Boolean operators: “Water quality monitoring” AND (“citizen science” OR “community-based monitoring” OR “community participation” OR “community-based participatory research” OR “participatory action research” OR “Participatory monitoring”) and “Water quality monitoring” AND (“volunteer” OR “citizen scientist” OR “monitoring plan” OR “collaborative governance” OR “Water management” OR “Water governance”). This comprehensive search strategy aimed to capture relevant literature encompassing the diverse aspects of citizen participation in water quality monitoring and community-driven water management initiatives.

For the Spanish database Redalyc, Google Scholar tools were utilized as search support to refine the queries on the site: “Monitoreo de la calidad del agua” AND (“voluntario” OR “científico ciudadano” OR “plan de monitoreo” OR “gobernanza colaborativa” OR “Gestión del agua” OR “Gobernanza del agua”) site:redalyc.org, y “Calidad del agua” AND (“voluntario” OR “científico ciudadano” OR “plan de monitoreo” OR “gobernanza colaborativa” OR “Gestión del agua” OR “Gobernanza del agua”) site:redalyc.org. This targeted search strategy aimed to identify relevant Spanish-language literature within the Redalyc database, encompassing concepts related to water quality monitoring, citizen participation, and community-driven water management initiatives.

### Eligibility criteria

These publications addressed citizen participation and water quality monitoring using at least one of the participation methods described in Table 1 and at some levels of participation (collaboration, contributory, co-creation).

**Table 1:** Description of citizen participation methods.

Method of citizen participation	Description	Characteristics	Reference
**Citizen Science (CZ)**	Citizen science refers to the active involvement of the public in scientific research endeavors, where citizens collaborate with scientists or institutions in collecting and analyzing data.	Involves individuals without necessarily having scientific training	[2, 3, 8–10, 29]
		Increases the amount of data collected over a larger geographic area	
		Promotes public participation in science and decision-making	
**Community-Based Monitoring (CBM)**	It is a collaborative process where engaged citizens, government agencies, industry, academia, community groups, and local institutions collectively monitor, track, and respond to issues of common interest within the community.	Recognizes the central role of the community in generating and utilizing data	[7, 13, 23, 24]
		May encompass various monitoring activities, from environmental surveillance to public service assessment	
		Seeks to strengthen the community’s capacity to address local challenges and make informed decisions	
**Participatory Water Quality Monitoring (PWQM)**	Involves active community participation in monitoring and assessing water quality.	Involves community members in collecting water data	[12, 15, 26]
		Can address a wide range of aspects, such as water quality, availability, and sustainable resource use	
		Promotes environmental awareness and community responsibility	
**Participatory Action Research (PAR)**	It is a research approach that involves community members in identifying problems, formulating solutions, and implementing actions to address water-related issues.	Combines research and action to generate significant social change	[25, 31]
		Encourages active community participation throughout the research process	
		Aims for co-creation of knowledge and solutions between researchers and community participants	
**Participatory Community Monitoring (PCM)**	Similar to participatory water monitoring, it focuses on active community involvement in water monitoring and management.	Emphasizes direct community involvement in all stages of the monitoring process	[15, 33, 34]
		Facilitates the identification and resolution of local water-related issues	
		Fosters community autonomy and empowerment in water resource management	
**Community Hydric monitoring (CHM)**	Autonomous spaces from state and corporate action have become a strategy of resistance and political struggle for various communities—indigenous, Afro-descendant, peasant, urban groups—to confront states and companies by generating their own information.	It focuses on environmental monitoring, both the quality and quantity of water in specific areas.	[18, 35, 36]
		These strategies deploy different material and symbolic resources as well as various ways of acting and generating knowledge about the territory.	
		Contributes to sustainable management of water resources at the community level.	

Source: Authors’ own elaboration

**Fig 1:**
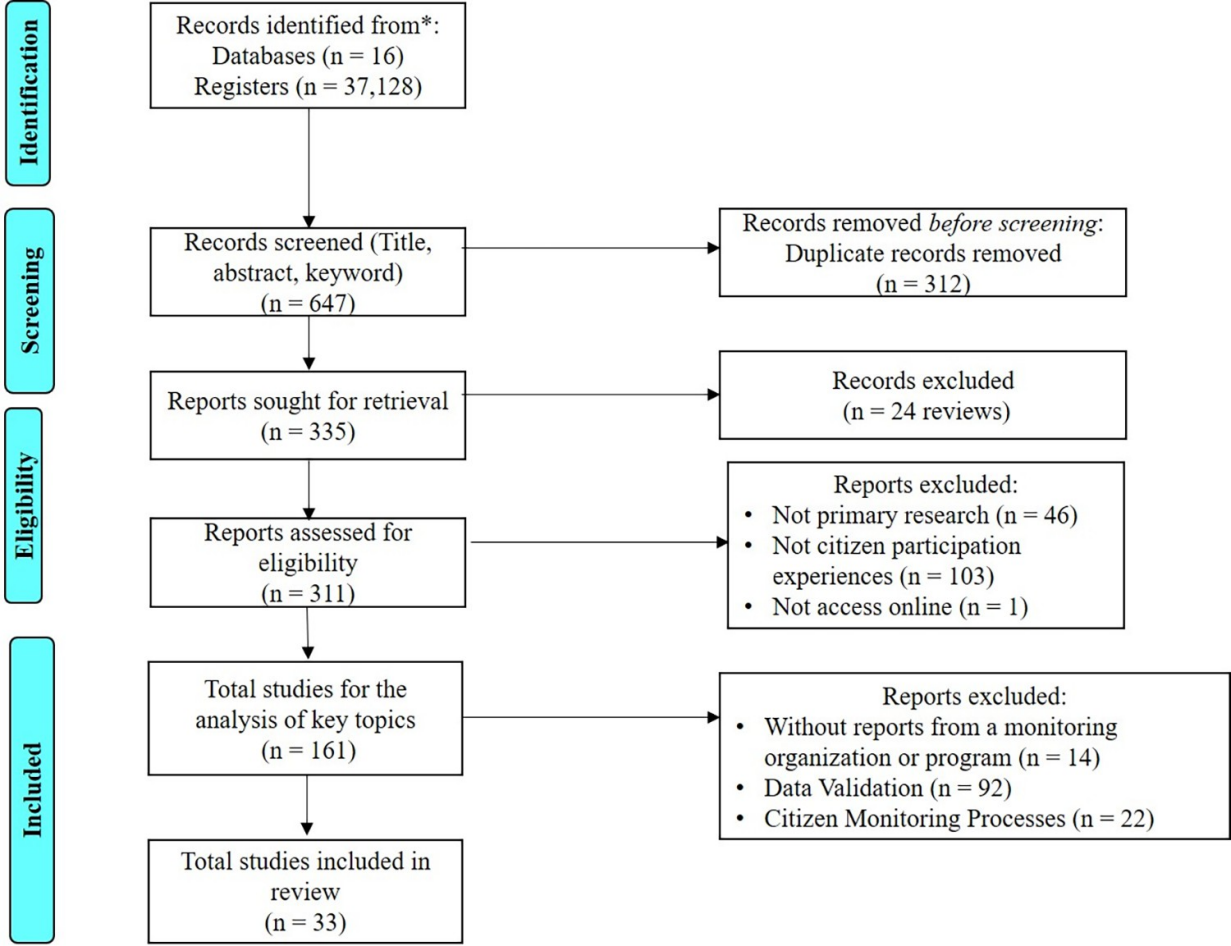
Publication Selection following the PRISMA 2020 guidelines.

Theses, reports, conference proceedings, popular science articles, informative notes, and opinion pieces were excluded from consideration. Publications detailing citizen monitors experiences gathered through surveys, interviews, focus groups, and discussion forums on organizational processes and the role of participants roles were considered. Duplicated documents (312 out of 647) and review or overview publications (24 out of 335) were omitted (Fig 1).

### Data extraction strategy

An analysis of key themes was conducted on the initial set of 161 articles to identify the method of citizen participation employed., the type of water source monitored (surface, groundwater, and/or drinking water), and the geographical location (by continent and country) to identify differences between the Global North and Global South. Based on this discern differences between the Global North and Global South. Based on this analysis, 33 studies were selected for further examination, as they presented at least one instance of community involvement utilizing citizen water monitoring (CWM) in any of its modalities.

### Synthesis method

A detailed review of the 33 case studies was undertaken, identifying the type of initiative, level of involvement by citizen scientists (volunteers), barriers or limitations faced by the project or program, funding sources, degree of transformation achieved, duration of the exercise, and challenges encountered. The findings are presented through tables categorized by global location and practices that strengthen community water organization structures.

### Publication bias assessment

The process of identifying, selecting, and extracting data from the documents was conducted in triplicate, with co-authors participating in the validation and analysis of each article to limit bias. The studies were weighted by each co-author as follows: "0" if the publication did not present evidence of being related to topics of water quality monitoring and citizen participation, "0.5" if the publication documented topics of water quality monitoring and citizen participation or community organization, but the journal did not meet quality requirements or provide open access, "1" if the publication focused on community organization, water quality monitoring, underwent peer review for publication, and was openly accessible. The weights were summed, and documents with a cumulative score of "3" were selected (161 out of 311 = 51.8% acceptance rate). Investigations detailing experiences of volunteers or community organizations in water quality monitoring were discarded if their method of citizen participation was not clearly defined, as well as publications that presented water quality monitoring data without detailed accounts of citizen participation (128 out of 161 = 79.5% rejection rate). Ultimately, the selected publications were those that clearly presented results of a community organization’s participation in water quality monitoring, applying at least one method of citizen participation (33 out of 161 = 20.5% acceptance rate).

Among them are the works of Goofried [35],Godfried [36],and Ulloa [18], which address the same cases with changes in the analytical approach. On the other hand, Kinchy [37] and Kinchy [38]mainly present survey results on NGO efforts in data recovery to measure the impact of fracking on water sources, without specifically focusing on a water community organization or monitoring program; a similar situation is found in Kerr’s work [39]). The inclusion of all six publications is considered relevant because they document various situations related to the success of programs and strategies implemented to strengthen the decision-making capacity of different water community organizations through water quality monitoring.

## Results

### Synthesis of selected studies

Among the 161 selected articles, citizen science emerged as the primary method of citizen participation, being mentioned in 55.6% of cases (Table 2). Community-based monitoring was reported to a lesser extent at 18.2%, followed by participatory monitoring at 13.9%, and participatory action research at 4.8%. Less common were instances of community water monitoring (4.2%) and community water monitoring (3.2%).

**Table 2:** Summary of articles by citizen participation method and type of water monitored.

Type of water	Citizen Participation Method						Total*
	Citizen Science	Community-Based Monitoring	Participatory Water Quality Monitoring	Participatory Action Research	Participatory Community Monitoring	Community Hydric monitoring	
**SW**	89	25	20	8	6	3	151
**GW**	7	4	2	1			14
**SW & GW**	3	3	2			3	11
**DW**	5	1	1	0			7
**SW, GW & DW**			1		1		2
**SW & DW**		1			1		2
**Total** *	104	34	26	9	8	6	187

*Mentions in the article

SW: Surface water; GW: Groundwater; DW: Drinking water or tap water (does not specify the water source).

**Fig 2:**
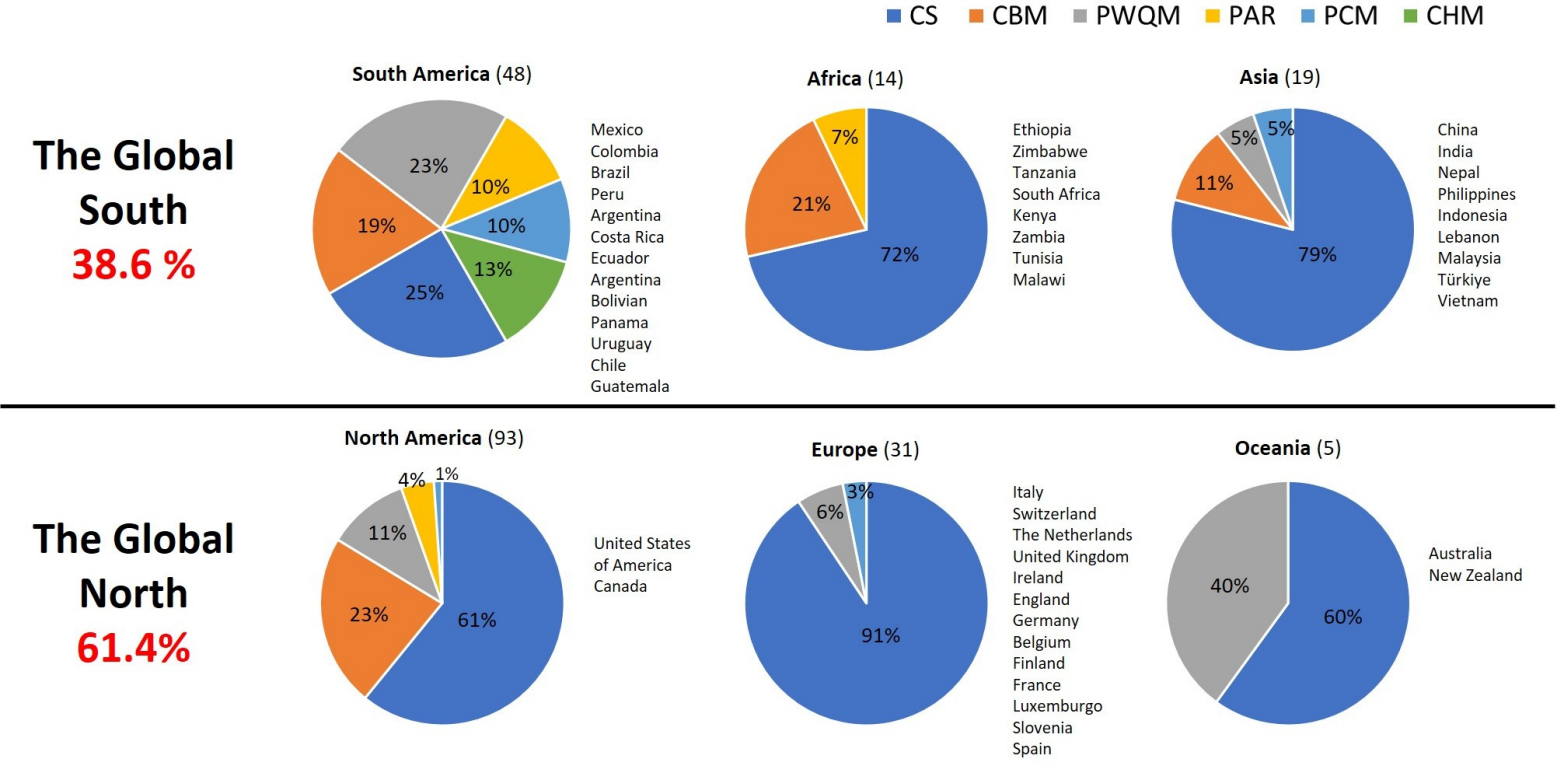
Citizen participation method by region and continent.

The primary water source monitored was surface waters (80.7%), with citizen science being the predominant method employed. Groundwater quality monitoring accounted for a smaller proportion (7.5%). Notably, few studies (5.9%) reported data on tap/drinking water without specifying whether the water originated from groundwater or surface sources. Additionally, it was identified that 61.4% of the exercises were concentrated in the Global North, primarily in the United States and Canada, in contrast to the Global South, which accounted for only 38.6% of the reviewed exercises (Fig 2).

### Contrast the experiences between the North and South Global

In the Global North, participants are referred to as volunteers, as their main task is to collect data, mostly without financial compensation, and in their free time. Their purpose is limited to establishing a baseline and continuing data generation over time [39–41], to increase public awareness or contribute to scientific knowledge [31]. An average of 25 volunteers per monitoring program has been recorded, ranging from 10 to 100 volunteers; however, larger groups often consist of students and academics using citizen science as an educational exercise [31, 39].

On the other hand, the Global South is characterized by initiating monitoring or community-based monitoring projects, where citizens determine sampling points and the use of data. In these exercises, the purpose is for participants to recognize the contamination risks of their water sources and engage them in informed decision-making in local water management [11, 23]. The number of participants and the project’s continuity are conditioned by the available funding from the organizing institution [often public universities] to purchase reagents, sampling materials, and transportation to reach sampling sites [42–45].

In contrast to the Global North, monitoring programs in the Global South are led by NGOs that manage grants from the government or receive support from international organizations such as LakeWash, ALLARM, HSBC, or WFF [46–48]. Notably, in the specific case of the United States, water quality monitoring programs experienced a boom in the 1980s. Faced with a lack of technical staff and data on the environmental conditions of surface waters, the Environmental Protection Agency (EPA) promoted water quality monitoring programs in different states. The EPA funded the purchase of kits, provided training, *facilitated* sample collection, and implemented other initiatives, to raise awareness about the state of water resources [40, 49, 50]. However, using subsequently faced data to influence public policy was not a consideration, and these programs have subsequently faced face budget cuts or cancellation [37, 51].

A significant strength of the Global North has been the establishment of numerous NGOs that focus on the conservation and restoration of natural resources. In recent years, there have been shifts in monitoring objectives for organizations located in New York and Pennsylvania, USA, in response to the recent expansion of natural gas extraction in the northeastern United States. This a process that utilizes new techniques (often called “fracking”) that pose a risk of unintended water contamination [31, 37]. Monitoring groups believe that maintaining a record of water quality data can aid in highlighting pollution problems as they emerge or in monitoring the activities of companies involved in the extraction process [37, 38].

In contrast, of exercises, such as inspections, are entirely rejected in the Global South. In countries like Peru, Argentina, and Colombia, where open-pit mining has led to multiple contamination issues, community water monitoring emerged as a strategy for defending the territory due to distrust in the data generated by mining companies and public institutions [35, 52, 53]. Despite the professionalization of community water monitors through certification processes with international organizations like Global Water Watch (GWW), states are unaware of local forms of knowledge generation related to water management and practices [18, 35].

### Community water organization structures and strengthening practices

#### Community water organization structures

A collective entity could serve as the main bridge to improve water access at the community level, as support for the entity and its initiatives stems from the recognition of the majority of organized community members [54]. For this review, community water organization structures, for this review, are acknowledged as any form of citizen organization, whether legally constituted or not, whose members are recognized by the community. These organizations have the capacity to collect and analyze data on water quality for knowledge generation and use in local decision-making. They are also engaged in environmental advocacy to protect water sources for human consumption and use. Based on the types of actors involved, three types of community organization structures were identified: 1) Community groups, comprised of community members, neighbors, farmers, and all individuals within a community; 2) Associated groups, formed by community members and representatives of NGOs, academics, students, or researchers; and 3) Institutional groups, primarily composed of government representatives, followed by NGOs, academics, and occasionally, citizen representatives (Table 3).

**Table 3:** Type of community water organization structures.

Name	Group Type	Location	Citizen Participation Method	Water Type	Reference
***Monitoring Programs***					
Lay Monitoring Program on Lake George (NY)	Community	United States of America	PWQM	SW & GW	[40]
Georgia Adopt, A-Stream & University of Rhode Island Watershed Watch (URIWW)	Community	United States of America	CS	SW	[51]
Citizen’s Water Quality Testing (CWQT) Program in New York City (NYC)	Institutional	United States of America	PWQM	SW	[12]
Waterlogged	Institutional	Canada	PCM	SW	[22]
Bajo Balsas program	Institutional	México	CBM	SW	[23]
Observatory of Water and Global Change (OACG) of the University of Costa Rica	Institutional	Costa Rica	PAR	SW	[25]
Florida LAKEWATCH Program	Institutional	United States of America	CS	SW	[46]
Illinois Volunteer Lake Monitoring Program	Institutional	United States of America	CS	SW	[50]
MIT & Sipayik researchers	Institutional	United States of America	PWM	SW, GW& DW	[15]
Training and Intervention Group for Sustainable Development (GRUFIDES) and Engineering Without Borders (ISF) & Other participants [Boards of Canal Irrigators, Users, and Service and Sanitation Administrative Boards (JASS). Representatives of the Regional Government of Cajamarca, Universidad Privada del Norte and National University of Cajamarca (UNC)]	Institutional	Peru	PCM	SW	[53]
The name of a community organization, institution, or program does not provide specificity.	Institutional	United States of America	PWQM	SW	[39]
IMALIRIJIIT -"Those who study water" en Inuktitut	Partnership	Canada	CBM	SW	[55]
Proyecto Tracking Change (Participants: Traditional Knowledge and Strengthening Partnerships Steering Committee (TKSPSC); Inuvialuit Joint Secretariat—Fisheries Joint Management Committee; Mikisew Cree First Nation; Gwich’in Renewable Resources Board; Sahtú Renewable Resources Board; Nacho Nyak Dun First Nation; Dehcho First Nations; Łútsël K’é Dene First Nation; Dena Kayeh Institute; Akaitcho Territory Government; Treaty 8 Tribal Association of British Columbia; Treaty 8 First Nations of Alberta; Prince Albert Grand Council)	Partnership	Canada	CBM	SW	[24]
Citizen Science Leaders (CSLs), Hongkong and Shanghai Banking Corporation (HSBC) staff participated	Partnership	China	CS	SW	[56]
Alliance for Aquatic Resource Monitoring (ALLARM)	Partnership	United States of America	PAR	SW	[31]
Clean Annapolis River Project (CARP)	Partnership	Canada	CS	SW	[43]
Alliance for Aquatic Resource Monitoring (ALLARM)	Partnership	United States of America	CS / PWQM	SW & GW	[57]
The name of a community organization, institution, or program does not provide specificity.	Partnership	United States of America	CS	SW	[37]
The name of a community organization, institution, or program does not provide specificity.	Partnership	United States of America	CS	SW	[38]
***Organization Figures***					
Association of Environmental Watchers and Monitors of Espinar (AVMAE)	Community	Peru	CHM	SW	[18, 35, 36]
Assembly Jáchal Not to be Touched (AJNST)	Community	Argentina	CHM	SW	[18, 35, 36]
Watch Committee (Comité de vigilancia)	Community	Mexico	CS	SW	[54]
Community Water Monitoring Network of the Monarch Butterfly Biosphere Reserve (Red de Monitoreo Comunitario del Agua de la Reserva de la Biosfera Mariposa Monarca)	Community	Mexico	CBM	SW	[58]
Friends of the Pixquiac River from the Neighborhood Association of Pixquiac-Zoncuantla (AVPZ)	Community	Mexico	CBM	SW	[59]
Union of Peasant and Indigenous Organizations of Cotacachi Canton (UNORCAC)	Community	Ecuador	CBM	SW & GW	[60]
The Jangas Committee	Institutional	Peru	PWQM	SW	[52]
Nature Conservation Center (NCC), of the American University of Beirut; & Water Committee at the Village	Partnership	Lebanon	CBM	GW	[11]
Boca Itata Neighborhood Council (Consejo Vecinal de Boca Itata,Participants: University of Alberta and 12 projects led by indigenous groups)	Partnership	Chile	CBM	GW	[45]
Community Based Environmental Monitoring Network (CBEMN)	Partnership	Canada	CS	SW	[43]
Network for monitoring the effect of glyphosate use on water quality (RMCA)	Partnership	Argentina	PAR	SW	[32]
Community Water Monitoring Network of the Monarch Butterfly Biosphere Reserve (Red de Monitoreo Comunitario del Agua de la Reserva de la Biosfera Mariposa Monarca)	Partnership	Mexico	CBM	SW & GW	[61]
Mountain Ecosystems Laboratory (LEM) of the Faculty of Sciences of the National Autonomous University of Mexico (UNAM) and organized community groups (Asociación de Comerciantes Unidos de los Dinamos, AC; the Magdalena River Basin Committee; the Forest Patrol; the Fire Brigade E-12 of the Magdalena Contreras Delegation; and two groups dedicated to ecotourism, Atlitic Nature Tourism and Atlitic Human Development in Contact with Nature)	Partnership	Mexico	PWQM	SW & GW	[62]
Water Committee (Comité del Agua)	Water Committee (Comité del Agua)	Ecuador	PWQM	SW & GW	[17]

#### a) Community groups

In Peru, the Committee for Participatory Environmental Monitoring and Surveillance- CMVAP and the Association of Environmental Monitors and Watchers of Espinar (AVMAE)emerged to document, with scientific data, the environmental impacts experienced by water sources. Their goal is to generate independent knowledge and use the data in dialogue and advocacy processes with mining companies and the government. They aim for the results to be recognized as a valid source of environmental information by various sectors of the government and society [36]. In a similar situation is the Assembly Jáchal Not to be Touched (AJNST), in Argentina, which has also taken direct actions such as (mobilizations, permanent installation of a tent in front of the municipal headquarters, performances, graffiti, and roadblocks). Furthermore, they have pursued legal actions including judicial complaints against officials and the company, presentation of ordinances, requests for popular consultations, and others; to improve access to and protection of water from the impacts of mining [31, 35].

#### b) Partnership groups (mixed)

Collaboration between community organizations and scientists within the framework of citizen science can yield significant mutual benefits, such as sharing resources, contextual knowledge, technical expertise, and common interests in monitoring, management, and environmental protection [22]. Examples of this are global monitoring programs like Global Water Watch (GWW), FreshWater Watch (FWW), and the Alliance for Aquatic Resource Monitoring (ALLARM). Additionally, local-level programs offer instances of such collaboration, including IMALIRIJIIT—meaning "Those who study the water" in Inuktitut [55], an environmental education program conducted with young people from the Inuktitut nation; water monitoring programs such as Georgia Adopt, A-Stream and the University of Rhode Island Watershed Watch (URIWW).

ALLARM, based at Dickinson College in Carlisle, Pennsylvania, and Nature Abounds, which supports efforts in the Allegheny Mountains, have developed community monitoring programs in consultation with representatives from state agencies [57]. These organizations serve as capacity-building and environmental advocacy entities by training volunteers, designing monitoring studies, selecting sampling sites, and identifying sources of funding. Their participatory approach fosters environmental stewardship and empowers local communities to protect their natural resources.

Partnerships between Non-Governmental Organizations (NGOs) and funding institutions in water quality monitoring programs often contribute to their success, as exemplified by the case of Ecological Restoration and Development A.C. (REDES) a Mexican NGO that trains volunteers to measure water quality in the peri-urban Xochimilco wetlands. From 2013 to 2016, REDES recruited volunteers from various workplaces and trained them as part of the Global Water Program, a collaborative initiative funded by the Hong Kong and Shanghai Banking Corporation (HSBC) and implemented by FreshWater Watch (FWW) and the Earthwatch Institute in Mexico [63]. This multi-stakeholder approach leverages the expertise of NGOs, corporations, and volunteers, fostering environmental stewardship and generating valuable data for wetland conservation efforts. Pérez-Belmont et al. [63] reported that program volunteers acting as citizen scientists, successfully generated a significant amount of physicochemical and microbiological data that proved invaluable for assessing water quality during dry and wet periods. Through their efforts, they were able to establish connections between the effects of intensive and semi-intensive agriculture on water quality. Moreover, the volunteers exhibited a notable shift in environmental awareness, comprehending the extent of anthropogenic impacts on the ecosystem, such as the use of inorganic fertilizers. As a result of this increased awareness, some volunteers continued their involvement in the monitoring program, while others began actively supporting and promoting agroecological farming practices as a more sustainable alternative to conventional agriculture.

Perevochtchikova et al. [33] present a case study of community participation in water quality monitoring in San Miguel and Santo Tomás Ajusco, Mexico City, in during 2015–2016. At the outset, community members were strategically selected and trained as citizen monitors based on several criteria: available project budget, willingness to engage in voluntary work without financial compensation, ability to invest time in training, and commitment to conducting monthly monitoring over the next one to three years.

#### c) Institutional groups

These groups are characterized by comprising representatives from government institutions directly involved in water management and governance, effectively serving as trained decision-making bodies. As demonstrated in the work conducted by Re et al. [17], a Water Committee based on participatory monitoring and composed of these decision-making institutions can play a pivotal leadership role in managing conflicts that may arise between different institutions or with civil society. This approach not only enhances the legitimacy and transparency of water management but also ensures that the diverse interests and perspectives of various stakeholders, including civil society, are taken into consideration.

Godfried et al. [35] emphasize that officials in the Peruvian government give validity to environmental monitoring results only when generated by qualified technical personnel using standardized equipment and procedures, meaning that data generation is solely the responsibility of the state. On the municipal level, the state conducts participatory monitoring where various actors, primarily mining companies, can participate. However, state institutions such as ANA or OEFA do not make monitoring results public, despite Peruvian law requiring it. In instances where information is published, it fails to capture the complexity of the monitored ecological systems or the perceived impacts on society.

#### Strengthening practices

The strengthening practices implemented by the community organizations identified are described in Table 4:

**Table 4:** Description of strengthening practices implemented by community organizations.

Strengthening Practices	Description
Establishment of common objectives and signing of collaboration agreements among actors/participants	Members establish common agreements to define objectives, goals, and responsibilities that benefit collaborative work among participating parties, to prevent subsequent disagreements.
Professionalization of citizen monitors	It involves the certification and recertification of citizen monitors through continuous training by allied members, using standardized protocols and applying quality control measures in data collection methodologies.
Validation of data by certified laboratories	In addition to on-site monitoring, water samples are taken for analysis in specialized laboratories to verify the reliability of the data obtained in the field.
Constant feedback and integration of local knowledge	Individual or group work sessions help establish continuous improvement processes regarding activities carried out before, during, and after water quality monitoring.
Mutual recognition of work	Various activities aimed at valuing and recognizing the work of each team member, in order to maintain participant motivation.
Periodic delivery of results to local authorities	Delivering results to communities obeys the social mandate to avoid data extraction and the right to access information about water quality conditions.
Organization and participation in social activities	Creating spaces to socialize and strengthen trust among participants beyond academic work.

### Common objectives and signing collaboration agreements among actors/participants

Members engage in defining collective objectives that benefit collaborative work, such as data generation [33, 44, 58], determining the water quality of a river or drinking water source [59], measuring the degree of water source contamination [15, 62], assessing pollution risks and anthropogenic impacts [32, 61], among others. Furthermore, they clearly outline responsibilities, the intended use of data, visions, interests, knowledge, and the type of contribution expected from each participant. This proactive approach to aligning goals, roles, and expectations helps prevent potential disagreements later on [20, 62].

The Community Water Monitoring Network of the Monarch Butterfly Biosphere Reserve (RBMM), The participating actors and sectors agreed on that the importance of a letter of intent for collaboration, internal regulations, and the establishment of basic rules for selecting monitoring sites and activities, community involvement, and the dissemination of results to community representation bodies. These collaborative frameworks have facilitated cooperation, communication, and transparency among the parties [61]. Working in partnership brings additional advantages, such as more equitable long-term access to water through community agreements and norms, as well as recognition and support from the collective [54].

### Professionalization of citizen monitors

Also known as community monitors or citizen scientists, involves training participants by academics (universities) or international organizations like GWW to develop capabilities and skills in field methodologies for determining physicochemical, biological, and even microbiological parameters in situ (on-site). The training aims to ensure the quality of data monitors collected [18, 34, 36]. Using of standardized protocols and the applying quality controls in data collection methodologies [43] allow for maintaining a rigorous and continuous system of certification and recertification. This scheme helps to oversee in-situ measurement processes and the implementation and memorization of protocols, contributing to monitors developing a technical language for presenting arguments to government representatives and effectively detecting the presence of chemicals in water [36, 52].

### Validation of data by certified laboratories (universities

In addition to in situ (on-site) monitoring, researchers, academics, students, or hired volunteers collect representative samples from the sites sampled by citizen monitors less frequently. The purpose is to conduct physicochemical and microbiological assessments in laboratories equipped with specialized equipment to compare the results with the data obtained in the field [19, 22, 56].

### Constant feedback and integration of local knowledge

In a collaborative work system, it is necessary to establish continuous improvement processes to adjust data collection techniques, identify factors that jeopardize the ecosystem [62], and incorporate the historical memory of residents regarding events or changes that have altered the water resource quality [35, 55]. For instance, in the case of the IMALIRIJIIT program, Inuit knowledge was used as a tool to document land use, the ecology of animal and plant species, the biophysical processes of the river, and changes observed in the watershed [55], as the occupants of the territory experience the impacts of water resource contamination. Rio et al. [61] and Sasal et al. [32] applied social mapping methods (participatory mapping), where monitors identified sites with a higher risk of contamination or where they perceived those anthropogenic activities had a greater impact on the ecosystem. The goal was to determine sampling points for water quality monitoring.

### Mutual recognition of work

Partnerships with university experts can undermine citizens’ confidence in their own ability to produce credible results, and the academic focus on peer-reviewed publications could influence the goals and approach of follow-up work [36]. A common strategy is to issue participation certificates by educational or governmental institutions [58, 61, 62]. Gérin-Lajoie et al. [55] mention that recognition events and initiatives, such as Scientist for a Day and certificates motivate and reinforce the self-esteem of young individuals. Additionally, platforms like social media could enhance the reach of recognition.

### Periodic delivery of results to local authorities

Among the agreements, there is an emphasis on disseminating or delivering results to community members, which can be done directly to community leaders through a community assembly [61, 64] or made available on digital platforms [55, 65]. With government authorities, other strategies are employed, such as sending official letters with the data or convening meetings with representatives from the environmental or water sector of the locality [35, 61].

Ramos-Elorduy et al. [62] mention that monitors, community authorities, and local administration representatives are invited to a workshop for evaluation and results presentation at the end of each monitoring year. During this workshop, an executive summary of the results analysis and challenges is presented, questions are clarified, improvements are proposed, and community bonding is encouraged. Based on their experiences and perceptions of the problems, their causes, and possible solutions, attendees have identified areas of opportunity to increase the impact and dissemination of the process. Furthermore, this exercise has allowed the presentation of the working groups’ needs to the authorities.

### Organization and participation in social activities

Conducting field trips, social gatherings, or picnics are bonding activities that strengthen trust beyond academic work. They provide spaces to share experiences and perceptions about what is important to each participant beyond water quality data collection [62]. On the other hand, Gérin-Lajoie et al. [55] implemented various recreational activities between scientific workshops and sampling periods to promote intergenerational and intercultural exchanges (fishing, games, swimming, walks, boat trips, among others) and positive relationships between scientists and community members.

### Challenges for the consolidation of community organization figures

#### Transfer of responsibilities

Faced with the limited reach of government institutions to monitor water sources, citizen groups participating in voluntary water quality monitoring programs are responsible for generating on-site data and sending samples to the laboratory. In some cases, NGOs or associations produce reports interpreting the results-with the support of academics [65, 66]. These tasks are assigned in the legislation of each country, and it is the responsibility of the State to provide the funding, technical personnel, and infrastructure to carry them out. However, few groups receive financial support, tax benefits, or validation of their results.

#### Institutional legitimacy

The results of CWM programs are often only recognized as official if the State leads the monitoring. However, these evolving processes need institutionalization to maintain social legitimacy and political effectiveness in validating the generated data [18]. According to Godfried et al. [35], delegitimizing community knowledge has led to two dynamics. On the one hand, through social mobilization, communities reject information generated by the State and companies, because they do not consider it reflects the water-related issues experienced in the territory. On the other hand, it has led to the “professionalization” of community members who, with the support of NGOs, have been trained in dynamics related to water control. Community water organizations should establish inclusive governance frameworks that incorporate community monitoring into decision-making at local, regional, and national levels, thereby avoiding reliance solely on government institutions that fund projects to achieve their objectives [49].

#### Paid participation / Hiring of local coordinators

Citizen monitoring relies on the voluntary work and enthusiasm of the monitors; however, a loss of interest is observed throughout the exercise [67]. In the case of citizen science in the Global North, enthusiastic participation is sustained by students and retired individuals [38, 65], who invest their leisure time in data collection. Meanwhile, citizen scientists in the Global South maintain local monitoring as a tool for territorial defense and denunciation of the implementation of extractive practices and their socio-environmental consequences [18, 35]. Nevertheless, financial sustainability is still dependent on funds provided by NGOs.

#### Knowledge and traditional roles in communities

The lack of recognition of the knowledge of citizen monitors and land occupants is frequently mentioned by State authorities for discrediting data generated by the community despite validation from academic institutions [66]. On the other hand, the level of equitable involvement of women and men is limited [11], due to customs prevailing in rural and indigenous communities, where the role of women is confined to household chores [60, 63]. Similarly, in Colombian indigenous communities, there is a demand to understand the environmental aspects not only focused on water but also including all aspects, both ecosystemic and cultural, requesting an increase in monitoring points closer to their communities [35].

#### Monitoring of groundwater sources

The number of monitored sites is less than a quarter according to the analyzed publications Table 2), even though groundwater is the primary source of freshwater for human use and consumption. Establishing groundwater quality monitoring takes years; for this, it is essential to create commitment and motivation among participants to ensure the data collection process continues beyond the timelines of academic projects [44]. Community organizations represent the opportunity to increase the available data from these water supply sources.

## Discussions

### Sur Global vs Norte Global

Citizen science stands out for maintaining sample collection through “volunteers” for a long time and on a larger scale. In contrast, citizen participation is limited to measuring specific physicochemical parameters and sending samples to laboratories [49].

Water quality monitoring programs in the Global North help fill information gaps by generating datasets used for environmental monitoring [65]. However, they do not actively demand that their data be considered to modify or implement public policies focused on water resource conservation, as their role is limited to “supporting the government.” In contrast, monitoring in the Global South becomes one of the tools for territorial defense and denunciation of the implementation of extractive practices and their socio-environmental consequences [35]. The data are used to confront governments in dialogues, legal processes, marches, and collective demands by contrasting the data generated by government institutes and their data. This is done to influence regulations and public policies for the conservation, protection, and preservation of water sources and the surrounding ecosystems through active and informed citizen participation at the local level, as mentioned by Machado et al. [68].

### Figures of community organization and strengthening practices

Of the three identified groups, only the institutional group can make decisions to modify, generate, and implement public policies focused on water management. They keep the spaces for citizen participation limited to validating reports and already structured processes. As in the case of the “Water Committee” in Ecuador [17]; ANA and OEFA in Peru, which, in response to community demands, initiated water quality monitoring with citizen participation, but the results obtained were treated discreetly, meaning they are not published or shared with the public [35]. For the improvements and strengthening of community organization, it is necessary to create public policies supported by legal standards, mechanisms, plans, and programs that include citizen participation, as seen in the case of Uruguay [20].

As Cummins et al. [13] indicated, agreements among various actors are the main strength of association groups, as they are better prepared to meet long-term financing, participant, and consolidation needs. However, the distances traveled by specialized personnel can limit for the scope of citizen monitoring. Gérin-Lajoje et al. [55] mention that monitoring efforts may focus on communities with higher population density due to unequal distribution and financial constraints, which impact the amount of time monitors can dedicate to data collection.

On the other hand, community groups emerge from association groups once strengthening practices have improved their resource management and data collection capacities. However, they struggle to sustain monitoring activities in the long term due to financial constraints [34, 59, 62] and the invalidation of their data by the state [18]. Therefore, it is still inconceivable for a community organization to function without the results validation process, even if their data collection capabilities are recognized [11].

### Challenges

Previous research [7, 10] agrees that citizen groups of water quality monitoring require specialized support and mechanisms to prevent overburdening their responsibilities, but few mention the generation of compensation strategies [24, 33]. In this regard, providing symbolic or in-kind economic compensation (such as fuel, transportation, or food), especially when monitoring areas are distant and require monitors to spend a significant part of the day without direct economic benefits. Although verbal recognition within the community and through social media is not explicitly mentioned highlighted in the texts, these are effective ways to acknowledge collaborative work and individual participants. In terms of horizontality, recognizing organization members and allies as equals in various monitoring tasks facilitates communication, strengthens trust bonds, and values each party’s work.

On the other hand, the consolidation of community water organization structures is conditioned by a series of institutional, economic, and social factors summarized in the lack of recognition of generated data, thus resulting in limited impact on decision-making and policymaking based on citizen monitoring information [10, 15, 24]. Therefore, it is necessary to establish processes to legitimize community figures in national regulations to obtain funding for continued data generation.

## Conclusions

The results of this systematic review identified significant differences in the approach to citizen monitoring of water quality between the North and the Global South. In the North, particularly in the United States, Citizen Science prevails as the main method, while in the Global South, there is a tendency to favor Community-Based Monitoring, a more inclusive and participatory approach. A relevant finding is identifying three types of figures: community, partnership, and institutional, widely known as “Water Committees,” which play a fundamental role in the Global South. Among the practices that strengthen these committees stand out are, the professionalization of citizen monitors and the generation of collaboration agreements with various allies, such as researchers and public or private entities with share objectives.

On the other hand, considerable obstacles were identified, especially in the Global South, where regions are affected by large-scale open-pit mining projects. There is a pronounced institutional resistance to water quality data generated by citizen monitors in these areas. Advancement in this challenge requires-implementing monitoring programs with public funding and developing public policies that institutionally support these efforts. It is necessary to develop inclusive governance frameworks that integrate community monitoring into decision-making at the local, regional, and national levels. Additionally, it is necessary to consider investigating the social and cultural impact of water quality monitoring on the methods outlined within communities. This could include studying how community monitoring strengthens cultural identity, promotes social cohesion, and fosters empowerment within local communities.

Finally, this article emphasizes the importance of citizen involvement in water management and sheds light on regional differences. The findings contribute to understanding global dynamics in water management and provide valuable guidelines for policy formulation and effective practices. Community monitoring can solidify itself as a transformative tool that empowers citizens in water decision-making, overcoming the identified challenges.

## Supporting information

S1 Checklist PRISMA 2020 checklist. (DOCX)

S1 Table Academic repositories consulted. (XLSX)
